# Role of lncRNAs in the Development of an Aggressive Phenotype in Gallbladder Cancer

**DOI:** 10.3390/jcm10184206

**Published:** 2021-09-17

**Authors:** Pablo Pérez-Moreno, Ismael Riquelme, Priscilla Brebi, Juan Carlos Roa

**Affiliations:** 1 Department of Pathology, School of Medicine, Pontificia Universidad Católica de Chile, Santiago 8380000, Chile; pablo.perezm@uc.cl; 2 Institute of Biomedical Sciences, Faculty of Health Sciences, Universidad Autoónoma de Chile, Temuco 4810101, Chile; ismael.riquelme@uautonoma.cl; 3 Laboratory of Integrative Biology (LiBi), Centro de Excelencia en Medicina Translacional (CEMT), Scientific and Technological Bioresource Nucleus (BIOREN), Universidad de la Frontera, Temuco 4810296, Chile; priscilla.brebi@ufrontera.cl

**Keywords:** long non-coding RNAs, lncRNA, prognosis, gallbladder cancer

## Abstract

Long non-coding RNAs are sequences longer than 200 nucleotides that are involved in different normal and abnormal biological processes exerting their effect on proliferation and differentiation, among other cell features. Functionally, lncRNAs can regulate gene expression within the cells by acting at transcriptional, post-transcriptional, translational, or post-translational levels. However, in pathological conditions such as cancer, the expression of these molecules is deregulated, becoming elements that can help in the acquisition of tumoral characteristics in the cells that trigger carcinogenesis and cancer progression. Specifically, in gallbladder cancer (GBC), recent publications have shown that lncRNAs participate in the acquisition of an aggressive phenotype in cancer cells, allowing them to acquire increased malignant capacities such as chemotherapy resistance or metastasis, inducing a worse survival in these patients. Furthermore, lncRNAs are useful as prognostic and diagnostic biomarkers since they have been shown to be differentially expressed in tumor tissues and serum of individuals with GBC. Therefore, this review will address different lncRNAs that could be promoting malignant phenotypic characteristics in GBC cells and lncRNAs that may be useful as markers due to their capability to predict a poor prognosis in GBC patients.

## 1. Introduction

In the last decades, the study of aggressive malignant characteristics in cancer has involved mainly protein-coding RNAs (mRNA encoded from genes), which have been associated with different mechanisms that promote an aggressive phenotype in cancer cells [1]. However, in recent years a new group of molecules named non-coding RNAs (ncRNAs) has been described to play an important role in the development of cancer due to its implication in several malignant cellular processes [2]. These ncRNAs include microRNAs (miRNAs), small-nuclear RNAs (snRNA), small-nucleolar RNAs (snoRNAs), ribosomal RNAs (rRNAs), small-interfering RNAs (siRNAs), PIWI-interacting RNAs (piRNAs), and long non-coding RNAs (lncRNAs), all with different action mechanisms able to regulate gene expression within the cells in both homeostatic and pathological conditions [3].

LncRNAs are defined as RNA sequences of more than 200 nucleotides that typically do not possess functional open reading frames (ORFs) and most are transcribed by RNA polymerase II [4]. These transcripts act by epigenetically regulating the gene expression at post-transcriptional, transcriptional, translational and post-translational levels by forming structures of RNA:RNA, RNA:DNA, and RNA:Protein that allow them to participate in different cellular processes [5,6]. The expression of lncRNAs has been associated with different types of cancers by inducing the acquisition of more aggressive characteristics, such as higher tumorigenic capacity, higher metastatic capacity, induction of epithelial-mesenchymal-transition (EMT) features, drug resistance, and stem-like phenotype, all of them directly related to poor prognosis in cancer patients [7,8,9,10]. These characteristics are frequently observed in different cancers such as breast, lung, colorectal, and gallbladder cancer [3,11]. Specifically, in gallbladder cancer (GBC), it has been shown that the expression of certain lncRNAs can promote the development of malignant features in cancer cells, correlating with a worsening of clinicopathological characteristics. Examples of this are the ROR and UCA1 lncRNAs, which have been shown to promote greater proliferative and invasive capacity, correlating with a worse prognosis in GBC patients [12,13]. For this reason, this review will address different malignant phenotypic characteristics that may be induced by lncRNAs in GBC cells and that may be useful to predict a more aggressive tumor phenotype in patients.

## 2. Search and Selection of Literature

A comprehensive search of the literature was performed by authors using online databases including PubMed and Web of Science. The search terms were as follows: (lncRNA OR long noncoding RNA) AND (gallbladder cancer OR gallbladder neoplasia OR gallbladder carcinoma) AND (poor prognosis OR aggressive phenotype OR metastasis OR drug resistance OR stem OR tumorigenesis OR invasion OR Migration OR proliferation). 

**Figure 1:**
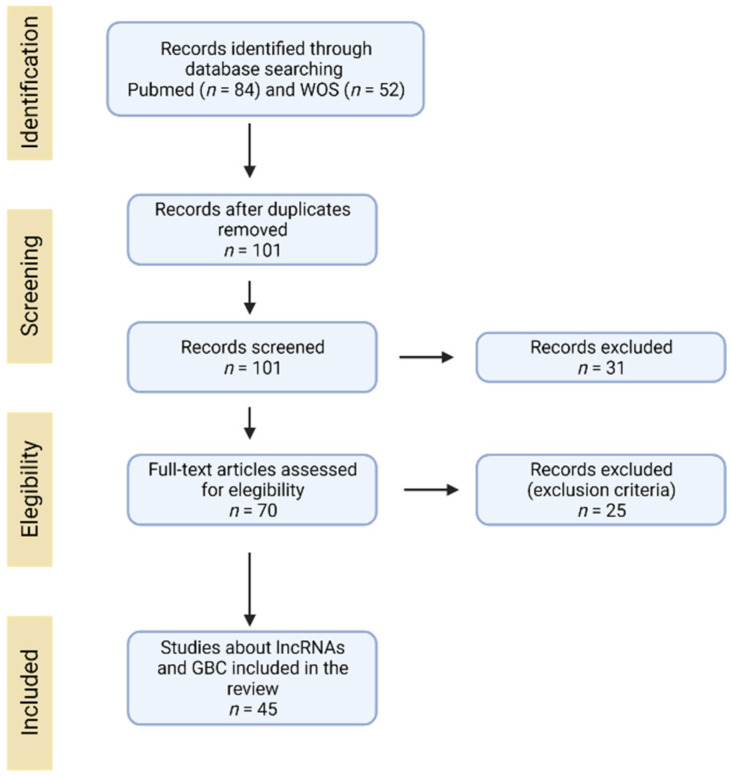
Search diagram used in the systematic review.

Selection criteria for lncRNAs in GBC were as follows: (1) lncRNA expression detected in tissues or serum from GBC patients; (2) lncRNAs related to aggressive phenotypic features in GBC (e.g., proliferation, invasion, epithelial-mesenchymal transition, tumorigenicity, migration, stemness, drug resistance and metastasis); and (3) lncRNAs related to some clinicopathological features (e.g., overall survival, TNM stage, tumor size, histological grade, distant metastasis, and lymphatic invasion). Those studies about lncRNAs not related directly to GBC were excluded from this article. The search diagram used in this article is shown in Figure 1.

## 3. Long Non-Coding RNAs: Mechanisms of Action

LncRNAs are divided into five broad categories according to the location relative to nearby protein-coding genes: intergenic, intronic, bidirectional, antisense and sense [14,15,16]. LncRNAs can exert their functions within the cells in both the nucleus and the cytoplasm. In the nucleus, lncRNAs actively participate interacting with *cis* or *trans* binding sites that induce the transcription or silencing of specific genes. In this regard, many lncRNAs can interact with the nuclear epigenetic machinery as the polycomb repressive complex 2 (PRC2) resulting in the control of gene expression [17,18,19]. For example, HOTAIR mediates the transcriptional silencing of HOXD locus via recruitment of the PRC2, promoting the subsequent invasion and metastasis in breast cancer [20]. Meanwhile, in the cytoplasm, these transcripts participate in the stabilization of mRNAs and the regulation of translation, for instance, by interacting with RNA binding proteins (RBPs) that lead to several alterations in the mRNA stability, splicing, protein stability and subcellular localization [21,22,23]. 

**Figure 2:**
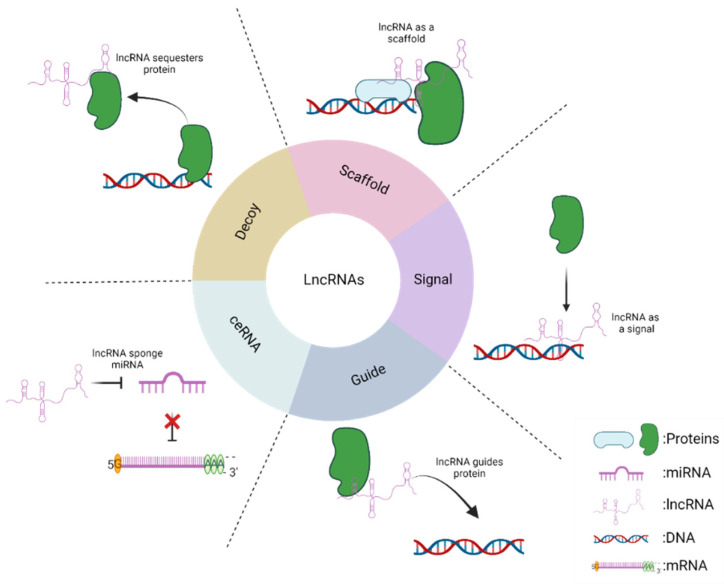
Scheme of the mechanisms of action of lncRNAs. This diagram shows the different forms in which lncRNAs can regulate different molecular processes. Decoy: lncRNA can sequester a protein of interest, interfering with its normal function. Scaffold: lncRNA can interact with different proteins allowing the formation of protein complexes. Signal: lncRNA acts as a tag for the recruitment of a protein to a specific site. Guide: lncRNA drives a protein of interest to its specific site of action. ceRNA: lncRNA acts as a competitive molecule that binds a miRNA sequence preventing the binding of this miRNA to its target (mRNA), allowing that the mRNA increases its stability and expression.

As mentioned above, lncRNAs are capable of physically interacting with different molecules, including DNA, RNA and proteins, generating complexes that allow the regulation of the function and expression of these macromolecules. Based on this, five main mechanisms in which this type of non-coding RNAs participates have been described [24]. First, lncRNAs can be signal molecules. This implies that they can increase or decrease against different stimuli determining a specific cellular context, for example, as indicators of transcriptional activity. In addition, this type of lncRNA can interact with DNA sites allowing the binding of different proteins, such as specific transcription factors, which allow regulation in the transcription of downstream genes. Second, lncRNAs can act as guides by binding to effector proteins that allow directing the target to a specific site. As an example, lncRNAs often interact with transcription factors that guide them to their target site to regulate gene expression. Third, lncRNAs can function as decoys since they can bind and sequester different target proteins preventing their normal function. Fourth, lncRNAs can function as scaffolds, because they can bind to proteins and mediate the formation of protein complexes regulating gene expression. Finally, lncRNAs can function as a competitive endogenous RNA network (ceRNA) since it has been shown that they can sponge miRNAs and prevent their binding to their mRNA target, causing an increase in the stability of the mRNA [25,26]. To improve understanding of these processes the mechanisms described above are summarized in Figure 2.

## 4. LncRNAs in Gallbladder Cancer

Gallbladder cancer has a mortality of 85,000 deaths worldwide and an incidence rate of 0.9 cases per 100,000 individuals [27]. The incidence of GBC varies among the different geographical areas of the world, showing a higher incidence among descendants of North and South American natives, and in several Asian countries [28]. The highest GBC incidence rate is found in Chile between individuals who descend from the Mapuche people, with 12.3 cases per 100,000 in men and 27.3 cases per 100,000 in women [28,29]. 

Unfortunately, as symptomatology is unspecific and routine biochemical assays are not accurate, GBC is usually diagnosed late, sometimes as an accidental finding in patients with cholelithiasis. Due to this late diagnosis, GBC is generally found in an advanced stage, which causes that these patients have a poor prognosis and short life expectancy [28]. For instance, the 5-year survival rate of GBC in advanced stages (T3 and T4 stages) is less than 5%, while if this cancer were detected in the initial stages (T1 stage) there would be an increase up to 75% in this 5-year survival rate [28]. 

The research about lncRNAs and their participation in the acquisition of a malignant tumor phenotype has evidenced a dramatic increase because a large number of them have been demonstrated to actively participate in several mechanisms that contribute to the progression of cancer [11]. Regarding this, the development of metastatic and tumorigenic characteristics is closely related to a more aggressive phenotype in cancer because these features provide cancer cells the capacity of expanding to other tissues and form new tumors, indicating a worse prognosis in cancer patients [1]. 

Due to the reasons previously stated, the search for new biomarkers that can help in the diagnosis and prognosis of the GBC cases is urgently necessary. In this search for more suitable biomarkers in the follow-up of GBC, lncRNAs seem to be molecules that are worth exploring in greater depth. 

Next, we will classify each lncRNA described according to its expression in GBC tissues.

### 4.1. Upregulated lncRNAs in GBC

Since the discovery of the first lncRNA in 1990 [30], many other lncRNAs have been described to date, using the latest technologies in molecular biology and due to large databases that have been able to provide a large amount of information on their molecular characteristics and biological functions in cancer [31]. Regarding the GBC, AFAP1-AS1, is a lncRNA that is overexpressed in GBC tissues and its expression levels are significantly associated with tumor size. The long-rank Kaplan—Meier analysis suggests that higher expression of AFAP1-AS1 is a poor prognosis factor in these patients. Functionally, the knockdown of AFAP1-AS1 may inhibit proliferation and invasion, and decrease Twist1 and Vimentin expression in GBC cell lines, indicating that AFAP1-AS1 may participate in cancer progression [32]. Similarly, ANRIL has been found upregulated in GBC tissues compared to adjacent normal tissues and has been also shown to increase proliferation and tumor size in a murine model, which is consistent with the correlation of its expression with overall survival in these patients [33].

CCAT1 has been described in different cancers, including gastric and colorectal cancer [34]. This lncRNA has been shown to be upregulated in GBC tissues compared with adjacent normal tissues. Furthermore, CCAT1 is more highly expressed in tumors in advanced stages than (T3 + T4) early stages (T1 + T2), and more highly expressed in tumors spread to lymph nodes (N1/2) compared with tumors localized only in the gallbladder (N0). CCAT1 expression is significantly correlated with tumor status, lymph node invasion and advanced tumor node metastasis (TNM) stage, suggesting that CCAT1 expression is related to poor prognosis in GBC. In addition, in vitro experiments show that the knockdown of CCAT1 decreases S-phase, invasion and tumor growth in vivo. Mechanistically, the authors propose that CCAT1 increases the expression of Bmi1 through competitively sponging miRNA-218-5p [35].

Along with this, current evidence has shown that those tumor tissues with a higher expression of cancer stem cell (CSCs) markers are prone to developing a greater tumorigenic and metastatic capacity and are directly associated with a worse life expectancy of patients [36,37,38,39,40]. Only one overexpressed lncRNA has been described in GBC patient tissues that promote the expression of markers associated with the stem-like cell population. DILC is overexpressed in gallbladder CSCs and GBC tissues and its knockdown decreased stem-like CD44^+^/CD133^+^ cell population and diminished sphere-forming capacity in GBC cells. Consistently, the expression of stemness-associated transcription factors and CSC markers (ABCG2, MDR-1, Oct4 and CD34) were also inhibited by DILC knockdown in GBC spheroids. Moreover, DILC knockdown decreased proliferation, migration and invasion in vitro, as well as tumor growth and CSC number in NOD/SCID xenograft model. In addition, the knockdown of DILC reduced metastasis capacity in vivo by activating Wnt/β-catenin pathway. This suggests that DILC promotes stem-like properties in GBC and can subsequently induce a pro-metastatic and pro-tumorigenic phenotype in this neoplasm [41]. 

Another lncRNA implicated in the development of aggressive features in cancer is DGCR5 [42,43,44,45,46]. This lncRNA is upregulated in GBC tissues and cell lines and has been associated with proliferation migration, invasion, colony formation, and tumor growth in vivo in GBC. Mechanistically, when DGCR5 is upregulated, a lower expression of ZO-1 and E-cadherin can be induced, whereas the expression levels of N-cadherin, vimentin, MMP-2, and MMP-9 are upregulated. Moreover, the MEK/ERK1/2 and JNK/p38 MAPK pathways may also be involved in the function performed by DGCR5 to induce invasion and EMT processes [47].

FOXD2-AS1 is another lncRNA that has been shown to induce proliferation, tumor growth, migration and invasion due to its effect on recruiting DNMT1, a methyltransferase that subsequently produces promoter methylation in the MLH1 gene, and consequently the inhibition of MLH1 transcription [48]. A study by Cai et al. identified around 457 overexpressed and 266 downregulated lncRNAs in doxorubicin (DOX)-resistant GBC cells. Among the overexpressed lncRNAs, GBCDRlnc1 seemed to be interesting because it was also found highly expressed in GBC tissues compared to adjacent noncancerous tissues. Kaplan—Meier analysis demonstrated that patients with GBCDRlnc1 overexpression have a significantly shorter overall survival than those with lower expression of this lncRNA, which was also correlated with histological grade and TNM stage [49]. In DOX-resistant GBC cell lines, the GBCDRlnc1 overexpression showed to be involved in an increased autophagy activity within the cells by enhancing the conversion from LC3-I into LC3-II and by inhibiting the ubiquitination of phosphoglycerate kinase 1 (PGK1). High GBCDRlnc1 levels were found in DOX-resistant GBC cells inducing a significantly greater resistance to doxorubicin, gemcitabine, and 5-fluorouracil in these cells. Therefore, this study suggests that chemoresistance observed in GBC cells may be related to the increased autophagic activity induced by GBCDRlnc1 [49].

Regarding GALM, this has been observed to be increased in tumor tissues from GBC patients and positively correlated with poorly differentiated cells, lymph node metastases, and TNM staging. Kaplan-Meier analysis revealed that elevated GALM expression is associated with reduced overall survival, suggesting that GALM expression is related to a worse prognosis in GBC. To observe the functional effect of GALM, the authors overexpressed this lncRNA in GBC cell lines, observing a greater migratory and invasive capacity in vitro. It is observed that the overexpression of GALM increased the levels of ZEB1, ZEB2, Vimentin and N-cadherin, suppressing the levels of E-cadherin. Accordingly, in vivo experiments demonstrated an increase in liver metastatic capacity, suggesting that GALM may promote EMT and metastatic ability in GBC. Notably, those cells that overexpressed GALM had a greater extravasation capacity, detected 48 h after intrasplenic injection of these cells in a mice model, suggesting that GALM promotes EMT and metastatic ability in GBC. Mechanistically, the authors showed that GALM functioned as sponges by competitively binding to members of the miR-200 family and binding to IL-1β mRNA, stabilizing it [50]. Similarly, HGBC has been involved in cell proliferation and the promotion of EMT and metastasis in GBC [24]. HGBC knockdown has shown a significantly decreased cell proliferation (over a 5-day culture), and a reduction in the colony formation ability in GBC cell lines. Similar effects have been observed when HGBC-knockdown GBC cell lines were injected subcutaneously into nude mice. The results showed that tumor volume and weight in mice injected with HGBC-knockdown cells were significantly decreased up to 30% compared to control. These data suggest HGBC has a potential role in the promotion of proliferation and tumor growth in GBC [24]. In addition, it has been shown that knockdown of HGBC reduces cell migration, expression of vimentin and N-cadherin, and decreased metastatic liver nodules after injection of cancer cells in the spleen in mice. This effect can be mediated by the binding among HGBC and an RNA-binding protein called Hu Antigen R (HuR). In addition, HGBC expression can be used as a progression and poor prognosis marker in GBC, particularly because the upregulation of HGBC has been positively associated with TNM stage and lymph node metastasis and has been significantly correlated with reduced overall survival [24]. 

HEGBC is another lncRNA that shares similar characteristics in GBC. HEGBC has been shown to increase their expression significantly in GBC cell lines and tissues compared to controls. Correlation analyses between the expression of HEGBC and clinicopathologic characteristics of GBC patients indicate that high HEGBC expression is positively correlated with lymph node metastasis and TNM stages. A Kaplan—Meier survival analysis has been shown that GBC patients with high HEGBC expression have worse survival than those with low HEGBC expression, suggesting that HEGBC expression is related to poor prognosis. In vitro, ectopic expression of HEGBC increased proliferation and migration, decreasing apoptotic capacity. In vivo experiments have shown that HEGBC overexpression in GBC cell lines significantly increased tumor growth in nude mice. The proliferation marker Ki-67 was higher in tumors that had HEGBC overexpression. Furthermore, GBC cells that stably overexpress HEGBC increased metastatic foci in the liver in nude mice. Mechanistically, it shows that HEGBC binds to the IL-11 promoter, increasing IL-11 transcription promoting an autocrine IL-11 signal, and activating the STAT3 signaling pathway. Furthermore, it has been demonstrated that STAT3 is also bound to the HEGBC promoter and activated HEGBC expression, suggesting that the effects induced by HEGBC are through a HEGBC/IL-11/STAT3 positive regulatory loop in GBC [51].

It has been shown that HOXA-AS2 is actively involved in human cancers [52]. About this, it has been shown a higher expression in GBC tumors and cell lines of HOXA-AS2. In addition, the overexpression of this lncRNA promotes proliferation, colony formation, apoptosis evading, migration, and invasion. Notably, the ectopic expression of HOXA-AS2 induces N-cadherin and Vimentin expression with the consequent decrease of E-cadherin, which suggests that HOXA-AS2 induces aggressiveness and EMT characteristics in GBC [53].

Another lncRNA is HOTAIR, which has been shown to be overexpressed in different cancers and to promote malignant tumor characteristics [54]. HOTAIR is overexpressed in gallbladder cancer tissues compared with adjacent non-tumoral tissues, indicating that HOTAIR is frequently upregulated on GBC. In addition, HOTAIR is more expressed in gallbladder tumors (T3 + T4) compared with tumors (T1 + T2). HOTAIR expression is more expressed in tumors spread to regional lymph nodes (N1) compared to primary tumors, suggesting that HOTAIR expression may be a progression and prognosis marker in GBC patients. Functionally, HOTAIR promotes proliferation and migration in GBC cell lines, correlating positively with the expression of c-Myc and negatively with miRNA-130a, which suggests that this mechanism may participate in cancer progression in GBC patients [54].

H19 is a lncRNA that has been expressed in different cancers in which it has been shown to participate in the acquisition of different oncogenic characteristics [55]. In GBC, H19 expression has been shown to be overexpressed in tumor tissue than in adjacent non-tumor tissue. Its expression is correlated with tumor size, lymphatic metastasis, and a worse prognosis in patients with GBC. Furthermore, H19 knockdown has been shown to decrease proliferation, tumorigenesis, migration, invasion, and EMT, by reducing Twist expression. Mechanistically, it was observed that overexpression of H19 in GBC cells downregulated miR-194-5p and markedly increased AKT2 expression, as well as enhanced the expression of miR-342-3p targeting FOXM1 through competitively sponging miR-342-3p, which suggests that the oncogenic function of H19 will be through both H19/miR-194-5p/AKT2 and H19/miR-342-3p/FOXM1 axes [56,57,58].

LINC01694 levels were also found remarkably elevated in GBC tissues, cell lines, and sera of patients with GBC. Patients with high LINC01694 values were more likely to develop stage III + IV and poorly differentiated GBC. Moreover, patients with higher LINC01694 levels had shorter total survival rates, being this transcript subsequently considered an independent factor in the prognosis of GBC patients. In vitro experiments evidenced that the LINC01694 knockdown effectively decreased proliferation and invasion compared to controls. In vivo analyses showed that the overexpression of LINC01694 significantly increased tumor growth in nude mice. Mechanistically, the higher expression of LINC01694 induced a reduction of miR-340-5p expression and, in consequence, a higher expression of SOX4. This result was confirmed in GBC tissues, which showed a positive correlation between LINC01694 y SOX4 expression but an inverse correlation with miR-340-5p expression. These data suggest that the aggressiveness induced by LINC01694 is via the LINC01694/miR-340-5p/SOX4 axis [59]. In a similar way, Loc344887 was found overexpressed in GBC tissues and cell lines compared to controls, and higher levels of Loc344887 were associated with larger tumor size. The Loc344887 knockdown was able to reduce the proliferative, migratory, and invasive features of GBC cells by decreasing the levels of Vimentin, N-Cadherin and Twist, and increasing the levels of E-cadherin, which suggests that Loc344887 promotes EMT and tumor progression in GBC [60].

LINC00152 has been widely described as an inductor of a tumorigenic phenotype in cells of several cancer types [61,62]. LINC00152 levels were found significantly higher in GBC tissues and in four human GBC cell lines compared to controls. The high LINC00152 levels were significantly associated with increased Ki-67-positive staining, a cell proliferation marker in tumors. In addition, the expression of LINC00152 was significantly higher in T3 + T4 compared to T1 + T2 tumors. Overexpression of LINC00152 significantly enhanced cell proliferation and the number of cancer cells in the S-phase in in vitro assays. This feature was confirmed in animal models where the high LINC00152 levels produced a greater tumor growth rate. Regarding the metastatic phenotype, LINC00152 expression levels were higher in metastatic lymph nodes than in primary tumors correlating positively with tumor status progression, lymph node invasion, TNM stage advancement and overall survival [63,64]. Moreover, the ectopic expression of LINC0052 promoted higher migration and invasion in vitro, as well as an increment in the number of peritoneal metastatic nodes in a murine model, accompanied by increased levels of Vimentin protein and decreased levels of E-cadherin protein. Mechanistically, LINC00152 can activate the PI3K pathway in GBC cells and can also act as a molecular sponge for miR-138, which directly suppresses the expression of hypoxia-inducible factor-1a (HIF-1a), suggesting that both the LINC00152/miR-138/HIF-1a axis and the activation of PI3K pathway by LINC00152 might be inducing GBC progression [63,64].

MALAT1 is one of the most studied lncRNAs in cancer [65,66,67] and has several oncogenic functions as modulates multiple signaling pathways involved in the enhancement of cell proliferation, metastasis, and invasion that commonly results in poor prognosis in cancer patients [68,69,70,71,72,73,74]. MALAT1 was found overexpressed in GBC tissue samples versus controls and its upregulation correlated positively with tumor size and lymph node metastasis, while also correlating negatively with overall survival [66,75,76,77,78]. The tumorigenic features developed by MALAT1 expression were evidenced by lower proliferation rate, lower colony formation and decreased tumor growth in in vivo models once GBC cells were treated with siRNAs against MALAT1 (siMALAT1) [66]. The metastatic features produced by MALAT1 are demonstrated in the fact that siMALAT1 significantly reduced the migration and invasion in GBC cell lines and produced a diminished expression of matrix metalloproteinase 9 (MMP-9), an enzyme involved in invasion by digesting the extracellular matrix. The confirmation of these results was performed in mice injected with siMALAT1 NOZ cells, which exhibited few metastatic peritoneal nodules at 8 weeks after inoculation, as well as a significant reduction in the levels of phosphorylated MEK1/2, ERK 1/2, MAPK, and JNK 1/2/3. These results suggest that the ERK/MAPK pathway participates in the MALAT1-induced metastasis of GBC cells [66]. Another probable mechanism by which MALAT1 may promote aggressive characteristics in GBC is regulating MCL-1 expression as a competing endogenous RNA for miR-363-3p [78].

MINCR expression is significantly elevated in GBC tissues, being associated with larger tumor size, lymph node metastasis, and shorter overall survival time, which suggests that MINCR is related to poor prognosis in GBC patients. Functionally, the knockdown of MINCR can reduce cell proliferation, migration, invasion, and EMT in vitro. As expected, tumor volume in vivo also significantly decreased in mice injected subcutaneously with GBC si-MINCR cells. This result can be explained because MINCR may be interacting with miRNA ribonucleoprotein complexes (miRNP) that also contain Ago2, and this interplay may modify the ability of miR-26a-5p to bind to EZH2, influencing its expression [79]. Similarly, NEAT1 is another lncRNA that has been found highly expressed in GBC tissues compared to controls. In addition, its knockdown decreases colony formation, migration, invasion in vitro, and tumor growth in vivo. The NEAT1 action mechanism probably involves serving as a sponge of miR-335 to subsequently provoke the increase of Survivin expression [80].

Recently, OIP5-AS1 has been described as a new lncRNA that participates in the acquisition of malignant characteristics in cancer [81,82,83]. In GBC cells lines (GBC-SD, NOZ, SGC996) this lncRNA has been shown to be significantly overexpressed. Furthermore, this OIP5-AS1 overexpression has been closely related to proliferation, migration, and invasion in GBC cell lines via reduction of miR-143-3p expression [84]. Another lncRNA significantly upregulated in GBC tissues is PVT1 in which expression was associated with advanced TNM stage and distant metastasis as well as correlated with a worse overall survival rate. Univariate and multivariate analyses showed that PVT1 was a potent independent prognostic indicator for GBC patients, suggesting that PVT1 expression is associated with poor prognosis in GBC patients. Additionally, PVT1 knockdown significantly inhibited cell proliferation, colony formation assay, migration, and invasion, inhibiting the expression of two matrix metalloproteinases, MMP-2, and MMP-9, suggesting that PVT1 can regulate EMT and cancer progression. Furthermore, the results demonstrated that these effects may be due to the up-regulation of HK2 by PVT1 through its competitive activity of endogenous RNA (ceRNA) on miR-143. However, other studies have been shown that these effects may be induced by other miRNAs, as miR-18b-5p and miR-30d-5p [85,86,87]. Another example is PAGBC, which is overexpressed in GBC tissues and is related with poor survival and advanced TNM stage, being considered as an independent prognostic factor for the overall survival of patients. This lncRNA promotes proliferation, colony formation, migration, invasion, and spleen metastasis in a mice model in GBC. This lncRNA can bind competitively to the tumor-suppressor microRNAs miR-133b and miR-511 activating the PI3K/mTOR pathway in GBC cells [88].

Another lncRNA is ROR, which has been involved in the acquisition of a more aggressive phenotype in GBC cases. ROR is upregulated in GBC tissues compared to matched normal tissues. The high expression of ROR has been found significantly associated with tumor size, lymph node metastasis and poorer overall survival time in GBC patients [13]. Also, ROR has been demonstrated to play an important role in cell proliferation, migration, and invasion in GBC cell lines. Silencing of ROR due to a siRNA against this lncRNA (siROR) induced a significant reduction in the number of cells in the S-phase and a decrease in the migration and invasion capacity of GBC cells [13]. To determine whether these aggressive characteristics are related to an EMT process, an siRNA against ROR was perform. The results showed that the mRNA expression of E-cadherin was significantly increased but Twist1 and Vimentin were markedly decreased in GBC cell lines, suggesting that ROR induces EMT in GBC cells [13].

SPRY4-IT1 is another non-coding RNA that is overexpressed in GBC cell lines and tissues versus controls. Its overexpression in GBC cell lines increases migration, proliferation, colony formation and invasion [89]. In a similar way, SNHG6 has been shown to be upregulated in serum from GBC patients and GBC cell lines compared to controls. This transcript has been closely related to poor prognosis because GBC has a correlation with grade of differentiation, TNM stage, tumor invasion, and location. The knockdown of SNHG6 may also decrease proliferation and invasion abilities in vitro, and reduced tumor growth in nude mice. Furthermore, these assays showed a significantly lower expression of N-cadherin, Vimentin and Snail while E-cadherin was significantly upregulated after transfection with siSNHG6, also indicating that SNHG6 can play a role in the development of EMT in GBC cells. This EMT process in GBC tumors may be triggered by SNHG6 via its downregulating effect on miR-26b-5p and the subsequent activation of the Hedgehog signaling pathway [90]. Another lncRNA is SSTR5-AS1 that has been involved in chemoresistance. SSTR5-AS1 has been found highly expressed in gemcitabine-resistant GBC cell lines. Results showed that upregulation of SSTR5-AS1 produced a decrease in apoptosis, specifically because the NONO/SSTR5-AS1 interaction prevents the degradation of NONO by the proteasome, thus suggesting that the apoptosis inhibition caused by SSTR5-AS1 is a signal of the development of drug resistance in GBC cell lines [91]. Moreover, SSTR5-AS1 was also found significantly upregulated in GBC tissues and cell lines compared to adjacent non-tumoral tissues, being associated with a worse overall survival rate in this cohort. Therefore, SSTR5-AS1 may be useful as a marker of prognosis marker in GBC patients [91]. Similarly, TUG1 has been found significantly overexpressed in GBC tissues and cell lines. Functionally, knockdown of TUG1 was able to significantly reduce proliferation and invasion in vitro, as well as inhibiting the Vimentin expression and upregulating the E-cadherin levels through a decrease in the miR-300 expression [92].

Another lncRNA involved in GBC progression is UCA1, whose upregulation has been observed in GBC tissues and GBC cell lines compared to controls. The UCA1 upregulation has been directly and significantly associated with certain clinicopathological characteristics of GBC patients including tumor size, lymph node metastasis, TNM stage, and poor overall survival time compared to patients with lower levels of UCA1 [12]. In tumor tissue injected subcutaneously in mice, the UCA1 overexpression also correlated with immunohistochemical expression of Ki-67, indicating that UCA1 may participate actively in proliferation and tumor growth processes [12]. In addition, UCA1 overexpression significantly promoted the cell migration and invasion of GBC cell lines by inducing the reduced expression of E-cadherin, and the increased Vimentin expression by Western blot. UCA1 expression was observed to be significantly upregulated after GBC cell lines were treated with TGF-β1, an activator o EMT process, which prompts that UCA1 can promote EMT in GBC through the recruitment of enhancer of zeste homolog 2 (EZH2) that subsequently induces the repression of p21 and E-cadherin in this malignancy [12].

TTN-AS and FENDRR are also found highly expressed in GBC tissues compared to controls. The knockdown of TTN-AS1 induced a decrease in migration and invasion capabilities by acting as a sponge to miR-107 and provoking the subsequent upregulation of HMGA1 [93,94]. Finally, CRNDE is a lncRNA that has not been described if its expression increases or decreases in patients with GBC. However, it has been observed that when a DMBT1 knockdown (CRNDE target as scaffold) is performed, the CRNDE expression increases. Furthermore, it has been observed that the expression of DMBT1 decreases in GBC tissues. Thus, these data suggest that CRNDE expression may be increased in GBC patients. Mechanistically, CRNDE acts as a scaffold for DMBT1, promoting migration and invasion through increased activity of the PI3K/AKT pathway [95].

For a better understanding, Table 1 resumes those lncRNAs that induce the acquisition of an aggressive phenotype in GBC.

### 4.2. Downregulated lncRNAs in GBC

As shown above, most of the lncRNAs described in GBC are overexpressed in these patients. However, there are lncRNAs that are also downregulated. An example of this is LET and GATA6-AS, which have been found downregulated in GBC tissues compared to non-tumor tissues. The low LET expression was correlated with less differentiated histology, greater invasion of lymph nodes, and advanced tumor stages. Moreover, multivariate analysis showed that LET expression was an independent prognostic indicator for metastasis and death, suggesting that the low levels of LET can serve as a prognostic indicator. Functionally, LET knockdown was able to increase the invasiveness and proliferative capacities under hypoxic conditions in GBC cells. On the contrary, LET overexpression increased apoptosis and suppressed proliferation and tumor growth in vivo. These results suggest that the lower expression of LET can participate in the progression of GBC [98]. Conversely, GATA6-AS expression was shown to progressively decrease as the gallbladder tumors proceeded to advanced stages, suggesting that GATA6-AS can serve as a marker of tumor progression. Furthermore, the overexpression of GATA6-AS was shown to significantly decrease the proliferative and invasive features in GBC cell lines. This effect can be explained by a decrease in the expression of miR-421 through TMP-2 [96].

Another lncRNA is MEG3 that it has been demonstrated that its expression is downregulated in GBC tissues compared with adjacent non-tumoral tissue. The low expression is related to poor prognosis, lymph node metastasis, and histological grade, suggesting that the low expression of MEG3 is related to poor prognosis in GBC patients. In vitro experiments showed that MEG3 overexpression significantly attenuated cell proliferation, colony formation and induce apoptosis in GBC cell lines. These results are reproduced in vivo since MEG3 overexpression decreases tumorigenesis and Ki-67 marker in the Balb/c nude mice model. In addition, MEG3 overexpression inhibited the invasion of NOZ cells with a significant upregulation of E-cadherin, accompanied by downregulation of N-cadherin and Vimentin. These data indicated that MEG3 inhibited cell invasion and EMT progression in GBC. Mechanistically, MEG3 promotes EZH2 degradation through its ubiquitination, regulating large tumor suppressor 2 (LATS2) as well as NF-κb pathway [33,99,100]. Similarly, GCASPC is a non-coding RNA that is downregulated in GBC tissues, being correlated with tumor size, advanced stage of disease, reduced overall survival (OS) and disease-free survival (DFS) rates. The ectopic expression of GCASPC decreased proliferation and induced significant G1-S arrest in GBC cell lines, while cell lines overexpressing GCASPC developed smaller tumors in nude mice, indicating that high expression of GCASPC reduces tumorigenesis in GBC. The authors mention that the probable mechanism will be via the inhibition of pyruvate carboxylase by the GCASP/miR-17-3p interaction proposing a new mechanism for the acquisition of a malignant phenotype in GBC [97].

In this review, the molecular and clinicopathological findings of lncRNAs described to date in GBC are summarized. Most of the lncRNAs studied in GBC have been selected due to being found overexpressed in GBC tissues and associated with malignant features that explain a worsening survival in GBC patients. Regarding this, a meta-analysis by Zhong et al., [62] describes similar findings to those reviewed in this work. The authors showed that overexpression of certain lncRNAs (e.g., MALAT1, MINCR, ROR, LINC00152, and AFAP1-AS1) can be used as potential predictors of worse survival or can be involved in the regulation of tumor size (e.g., AFAP1-AS1, MALAT1 and ROR). In addition, the overexpression of certain lncRNAs (e.g., LINC00152, HEGBC, MALAT1, and ROR and HEGBC) were found to be positively correlated with lymph node metastases, meanwhile, other lncRNAs such as PVT1 were correlated with a higher TNM stage. In contrast, Zhong et al. also described that the expression of LET and MEG3 was associated with a lower histological grade, lower TNM stage and absence of lymph node metastases, which is consistent with what has been described about the tumor suppressor role of these lncRNAs in other cancers [101,102]. Therefore, the inferences concluded in Zhong et al., corroborate the features described in this review.

For a better understanding of the clinical correlation of these lncRNAs, Table 2 resumes the correlation between lncRNAs and clinicopathological features in GBC.

**Figure 3:**
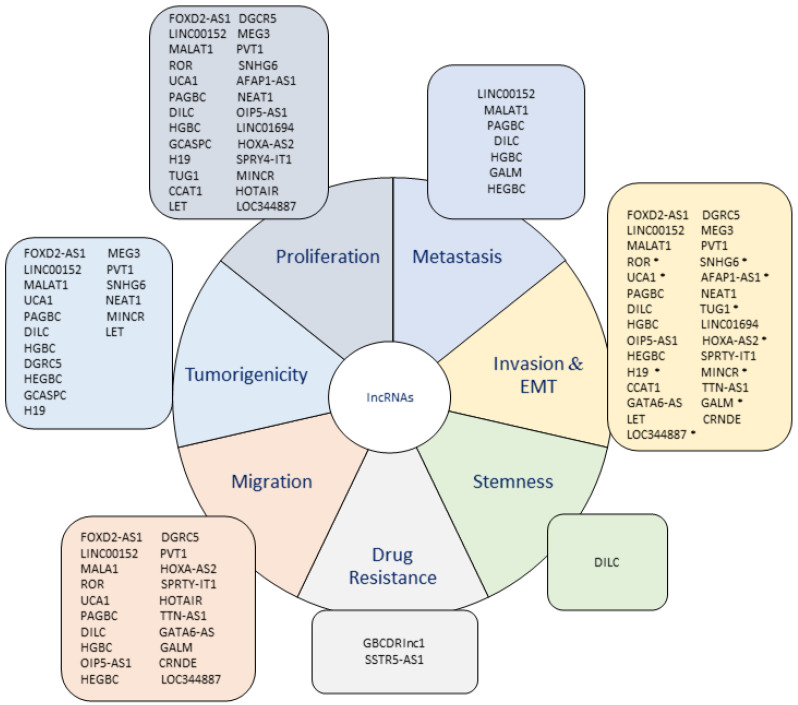
Role of lncRNAs in the aggressive phenotype in gallbladder cancer. LncRNAs associated with aggressive characteristics described in GBC. *: involved in invasion and EMT. lncRNAs without * are involved only in invasion.

Finally, Figure 3 summarizes all the features associated with an aggressive phenotype and its relationship with the different lncRNAs described to date in GBC.

## 5. Conclusions

The discovery of new cancer-related lncRNAs has been accelerated due to the development of high-throughput technologies. In addition, new cellular and molecular biology techniques along with new bioinformatic tools have been useful to describe the functional mechanisms of these lncRNAs in the acquisition of malignant phenotypic characteristics in cancer, such as GBC.

In this review, 30 lncRNAs were described as overexpressed in GBC patients (e.g., MALAT1, MINCR, ROR, AFAP1-AS1, PAGBC, LINC00152, UCA1, HEGBC, and PVT19) and were associated with worse clinical outcomes. Additionally, four lncRNAs were described as downregulated (e.g., LET, GCASPC, and MEG3), also acting as agents whose low expression promotes malignant tumor characteristics in these patients, worsening the overall survival (OS) in these patients. This suggests that lncRNAs can act as tumor suppressor or oncogenic transcripts, which account for complexity and the diversity of cellular processes in which they participate. Another finding is that one of the most relevant mechanisms of action of lncRNAs observed in this review are through ceRNAs (e.g., MALAT-1, PAGBC, LINC00152, MINCR, PAGBC, and PVT1), which is consistent with the mechanisms that can be described for lncRNAs that are upregulated in GBC, suggesting that lncRNAs act mainly as “sponging” against miRNAs, maintaining stability of oncogenic mRNAs and mainly inducing the activation of relevant signaling pathways widely described in cancer, such as PI3K/Akt, Wnt-β-catenin and MAPK.

It is known that the acquisition of a stem-like phenotype in cancer cells leads to the acquisition of an increasingly aggressive phenotype, which has been correlated with a worse prognosis in cancer patients. In this review, DILC was the only lncRNA described as a promoter of a stem-like phenotype in GBC cancer, which was related to increased tumorigenic and metastatic capacity. This data suggests that at least in GBC, there is still a need for more research on the role of lncRNAs in the acquisition of a stem-like phenotype, considering the clinical importance that this involves.

Clinically, the deregulation of these lncRNAs has been associated to poor prognosis evidenced by worsening of clinicopathological features. This correlation is consistent with the cellular phenotypic characteristics found in in vitro and in vivo models since it has been shown that the worsening of the patient’s clinical state is due to a greater aggressiveness acquired by cancer cells, in this case, attributed to the role of lncRNAs in cancer. Regarding this, it has been observed that tumor size has been correlated with greater tumorigenic capacity and metastasis (lymphatic or distant) with EMT and invasive capacity. Therefore, the need to use lncRNAs as prognostic or diagnostic biomarkers is becoming an urgent aspect to address.

One of the diagnostic methods that has been growing in recent years is liquid biopsy, which through different studies has demonstrated to be potentially useful for a great variety of clinical analyses, being used mainly for the detection of diagnostic or prognostic biomarkers in cancer patients due to its much easier sample collection. Regarding this, only two lncRNAs (SNHG6 and LINC01694) described in this review were detected in serum samples, which implies that more studies are still needed to determine the helpfulness of these methods as a routinary methodology clinically viable for the detection of lncRNAs in individuals with cancer.

In summary, many lncRNAs are aberrantly expressed in samples from GBC patients and are involved in different oncogenic processes in GBC. However, it is necessary to study the molecular mechanisms in which lncRNAs are involved and the potential utility of these transcripts as clinical biomarkers in GBC cases, more than simply establishing the differential expressions in this cancer.

## Figures and Tables

**Table 1:** Resume of lncRNAs related to an aggressive phenotype on gallbladder cancer.

Expression	LncRNA	Aggressive Phenotype	Mechanism	Ref.
Up-regulated	AFAP1-AS1	P, I, E	ND	[34]
	ANRIL	P, T	ND	[33]
	CCAT1	I, P	Increases BMI-1 expression through the sponging of miR-218-5p	[35]
	DILC	S, P, I, M, T, ME	Promotes Wnt/β-catenin pathway	[41]
	DGCR5	P, T, I, M, E	Decreases ZO-1 expression and increases MEK/ERK1/2 and JNK/p38 MAPK pathways	[47]
	FENDRR	ND	ND	[94]
	FOXD2-AS1	P, M, T, I	Promotes MLH1 methylation by recruiting DNMT1	[48]
	GBCDRlnc1	D	Increases autophagy activity by enhancing the conversion from LC3-I into LC3-II and by inhibiting the ubiquitination of phosphoglycerate kinase 1(PGK1)	[49]
	GALM	I, M, ME, E	Acts as a sponge by competitively binding to miR-200 family and binding to IL-1β mRNA	[50]
	HGBC	P, T, M, E, ME	Acts through the interaction among HGBC and HuR	[24]
	HEGBC	P, M, T, ME	Acts through a HEGBC/IL-11/STAT3 positive regulatory loop	[51]
	HOXA-AS2	P, M, I, E	ND	[53]
	HOTAIR	M, P	Increases c-Myc expression through the sponging of miR-130a	[54]
	H19	P, T, M, I, E	Acts through both H19/miR-194-5p/AKT2 and H19/miR-342-3p/FOXM1 axes	[56,57,58]
	LINC01694	P, T, I	Acts via LIN01694/miR-340-5p/SOX4 axis	[59]
	LOC344887	P, M, I, E	ND	[60]
	LINC00152	P, T, E, M, I, ME	Acts through LINC00152/miR-138/HIF-1a axis activating PI3K pathway	[63,64]
	MALAT1	P, T, M, I, ME	MALAT1 regulates MCL-1 expression as a competing endogenous RNA for miR-363-3p	[66,75,76,78]
	MINCR	P, M, T, I, E	Modulates the ability of miR-26a-5p to bind to EZH2	[79]
	NEAT1	P, M, I, T	Increases surviving expression and acts as a sponge of miR-335	[80]
	OIP5-AS1	P, M, I	Reduces miR-143-3p expression	[84]
	PVT1	P, M, I, E	Upregulates HK2 through its competitive endogenous activity on miR-143 as well as miR-18b-5p and miR-30d-5p	[85,86,87]
	PAGBC	P, M, I, ME	Binds competitively miR-133b and miR-511 activating PI3K/mTOR pathway	[88]
	ROR	P, M, I, E	ND	[13]
	SPRY4-IT1	P, I, M	ND	[89]
	SNHG6	P, I, T, E	Decreases miR-26b-5p expression and regulates Hedgehog signaling pathway	[90]
	SSTR5-AS1	D	NONO/SSTR5_AS1 interaction prevents the degradation of NONO	[91]
	TUG1	P, I, E	Decreases miR-300 expression	[92]
	UCA1	M, T, I, E	Recruits EZH2 and induces p21 repression	[12,77]
	TTN-AS1	M, I	Acts as a sponge to miR-107 and upregulates HMGA1 expression	[93]
Down-regulated	GATA6-AS	P, I	Decreases mir-421 expression through TMP-2	[96]
	GCASPC	P, T	Inhibition of pyruvate carboxylase by GCASPC and miR-17-3p	[97]
	LET	P, I, T	ND	[98]
	MEG3	P, T, E, I	Promotes EZH2 degradation regulating LATS2 and NF-κb pathway	[33,99,100]

ND: not described; P: proliferation; I: invasion; E: epithelial-mesenchymal transition; T: tumorigenicity; M: migration; S: stemness; D: drug resistance; ME: metastasis.

**Table 2:** Resume of lncRNAs expression related with clinicopathological features on Gallbladder cancer.

LncRNA	Overall Survival	Tumor Size	TNM Stage	Histological Grade	Distant Metastasis	Lymphatic Invasion	Ref.
AFAP1-AS1	Decrease	Increase	NS	NS	ND	Negative	[34]
ANRIL	Decrease	NS	NS	NS	ND	Negative	[33]
CCAT1	ND	Increase	Advanced	ND	ND	Positive	[35]
GBCDRlnc1	Decrease	NS	Advanced	Poorer	ND	Negative	[49]
GCASPC	Decrease	Increase	Advanced	ND	NS	Positive	[97]
GALM	Decrease	NS	Advanced	Poorer	ND	Positive	[50]
HGBC	Decrease	ND	Advanced	NS	ND	Positive	[24]
HEGBC	Decrease	Increase	Advanced	NS	ND	Positive	[51]
HOTAIR	ND	ND	Advanced	ND	ND	Positive	[54]
H19	Decrease	Increase	NS	NS	ND	Positive	[56,57,58]
LINC01694	Decrease	NS	Advanced	Poorer	ND	ND	[59]
LET	Decrease	Increase	Advanced	Poorer	ND	Positive	[98]
LINC00152	Decrease	Increase	Advanced	NS	ND	Positive	[63,64]
MALAT1	Decrease	Increase	NS	NS	ND	Positive	[66,75,76,78,103]
MEG3	Decrease	NS	Advanced	Poorer	ND	Positive	[33,99,100]
MINCR	Decrease	Increase	ND	ND	ND	Positive	[79]
PAGBC	Decrease	ND	Advanced	ND	ND	ND	[88]
PVT1	Decrease	NS	Advanced	NS	Increase	ND	[85,86,87]
ROR	Decrease	Increase	NS	NS	ND	Positive	[13]
SNHG6	ND	Increase	Advanced	Poorer	Increase	Positive	[90]
SSTR5-AS1	Decrease	ND	ND	ND	ND	ND	[91]
UCA1	Decrease	Increase	Advanced	NS	ND	Positive	[12,77]

NS: not significant; ND: not described; Advanced: stage ≥ pT2. The results: decrease, increase, advanced, poorer and positive are expressions statistically significant.
